# Incidental Learning of Gender Agreement in L2

**DOI:** 10.1007/s10936-017-9487-x

**Published:** 2017-04-13

**Authors:** Nadiia Denhovska, Ludovica Serratrice

**Affiliations:** 1 School of Psychology, University of Central Lancashire, Darwin Building, DB116, Preston, PR1 2HE UK; 2 School of Psychology and Clinical Language Sciences, University of Reading, Reading, UK

**Keywords:** Incidental learning, L2 grammar, Gender agreement, Working memory

## Abstract

Incidental learning of grammar has been an area of interest for many decades; nevertheless, existing research has primarily focused on artificial or semi-artificial languages. The present study examines the incidental acquisition of the grammar of a natural language by exposing adult speakers of an ungendered L1 (English) to the gender agreement patterns in Russian (a language that was novel to the learners). Both receptive and productive knowledge and the mediating role of working memory (WM) in learning were measured. Speakers of the ungendered language were able to successfully acquire receptive but not productive grammatical knowledge in a new language under incidental exposure. WM was engaged in production but not in a grammaticality judgment task in the incidental learning condition, indicating cognitive effort during knowledge retrieval.

## Introduction

Implicit learning refers to the human ability to derive information about the world in an unconscious, non-reflective way (Winter and Reber 1994), leading to implicit knowledge. In contrast, explicit learning is understood as a conscious process based on selective attention, leading to explicit knowledge (Leow 2000; Rebuschat and Williams 2012; Robinson 2005; Schmidt 1993; Williams 2005). Incidental learning conditions, however, are defined as learning environments in which learners are unaware of the underlying grammatical regularity. In studies employing such experimental conditions, during the training phase participants are usually asked to understand the meaning of sentential stimuli without receiving feedback on their performance, and without being informed about subsequent testing (Rebuschat and Williams 2012). In the present paper, we adopt these definitions of incidental and implicit learning.

Many essential skills, including language-related skills, are acquired incidentally, i.e., without conscious intention to learn and without awareness of the rules and regularities underlying the input. However, some scholars have postulated that procedural learning mechanisms are no longer available for the acquisition of L2 grammar in adulthood (Bley-Vroman 2009; Lenneberg 1967; Newport 1990; Ullman 2001, 2004). Therefore, if not acquired before the critical period ends, a dependency such as gender agreement represents a significant challenge for L2 learners, particularly when their L1 does not feature grammatical gender. L2 adult learners rarely attain native-like proficiency and have persistent difficulty in mastering grammatical gender (Lew-Williams and Fernald 2010).

Although numerous studies within the artificial grammar learning paradigm have demonstrated the potential for grammatical knowledge acquisition in adulthood (Leung and Williams 2011; Morgan-Short et al. 2010; Rebuschat and Williams 2012; Williams 2005), it remains unclear to what extent grammatical features in a novel, natural language can be acquired incidentally. In the present paper, we approach this language learning issue by exposing speakers of a language without grammatical gender to the noun-adjective gender agreement pattern in a natural language (Russian). In the following we present a brief discussion of the research on incidental learning and grammatical gender agreement. We then discuss our investigation targeting pattern acquisition by novice learners under an incidental learning condition and an explicit learning condition.

### Incidental Learning of L2 Grammar

Proponents of the Fundamental Difference Hypothesis posit that learning the grammar of a L2 is fundamentally different from acquiring L1 grammar. Consequently, the acquisition of L2 grammar is subserved by declarative rather than by procedural mechanisms (Bley-Vroman 2009; Ullman 2001). Deficit approaches claim that L2 grammar features that are absent in the learner’s L1 can be effortlessly acquired only before puberty (Tsimpli and Roussou 1991).

Thus, for a novice adult learner with an ungendered L1, acquiring the morpho-syntactic marking of gender agreement is a demanding task (Larsen-Freeman 2010). Priming studies have demonstrated that L2 learners show a lack of sensitivity to inflectional morphology during comprehension (Silva and Clahsen 2008). Production studies focusing on English–French and English–Spanish late learners have also found a high number of errors during the processing of gender agreement (Hawkins and Franceschina 2004). In contrast, representational accessibility approaches (Schwartz and Sprouse 1996) and cognitive approaches to L2 acquisition (Ellis 2002, 2006) predict the learnability of this structure (White et al. 2004; Leung 2005). Despite evidence from artificial language learning studies showing that adults can incidentally acquire new grammatical knowledge (De Graaff 1997; Reber 1967; Rebuschat and Williams 2012; Morgan-Short et al. 2010, 2012), studies of natural languages remain limited (Brooks and Kempe 2013; Godfroid 2016).

Some theoretical frameworks of implicit learning highlight the importance of associative mechanisms that facilitate the tracking of elements co-occurring in the input (Cleeremans et al. 1998; Johnstone and Shanks 2001; Knowlton and Squire 1994, 1996; Perruchet and Pacteau 1990; Saffran 2003; Saffran et al. 1997), and emphasize the human predisposition for pattern finding (Kirkham et al. 2002). Research indicates that learning of underlying grammatical structures may be based on the processing of specific individual items (Whittlesea and Dorken 1993) rather than on the immediate abstraction of a rule.

Research investigating the implicit/incidental learning of grammar has primarily focused on artificial languages (De Graaff 1997; De Jong 2005; DeKeyser 1995; Dienes et al. 1991; Hulstijn 1997, 2005; Knowlton and Squire 1994, 1996; Morgan-Short et al. 2010, 2012; Reber 1967; Rebuschat and Williams 2012; Williams 2005). Although the use of an artificial language facilitates control over confounding factors, it abstracts away from the mapping of form and meaning, which is one of the defining features of natural languages. In contrast, L2 acquisition studies that have focused on grammar learning have primarily utilized languages that were to some extent familiar to learners (Godfroid 2016; Lee 2002; Robinson 1996). The present study thus aimed to focus on the initial stages of the acquisition of gender agreement in both the receptive and the expressive domains while avoiding the limitations and constraints of some previous research (see Brooks and Kempe 2013 for a similar choice). First, by using a natural language, such as Russian, we aimed to provide learners with stimuli that are more ecologically valid than those used in artificial grammar studies, in which form is separated from meaning. Secondly, by choosing a language that was completely new to our participants, we controlled the amount of input addressed to the learners—unlike in previous studies where even beginner learners already had some familiarity with the L2. This allowed us to investigate incidental L2 acquisition at the very initial stages.

### Gender Agreement

According to Corbett (1991), the existence of gender is revealed by morpho-syntactic agreement. Compared to English, which does not mark grammatical gender on either nouns or adjectives (e.g., *red book*), Russian adjectives grammatically agree with nouns in their gender, case and number (Lorimor et al. 2008). Masculine nouns end with a consonant (e.g., *tsvetok* ‘flower’), feminine nouns usually end with –*a* (e.g., *shlyapa* ‘hat’), and neuter nouns usually end with –*o* (e.g., *yabloko *‘apple’). Because of the process of gender concord (Carroll 1999; Zagona 2002), the adjective changes its inflection in accordance with the noun’s gender, which dictates the variability in the inflectional pattern of the adjective (see Table 1 for the paradigm utilized in the present study). Thus, grammatical agreement is a mechanism that signals the relations of different linguistic items in a phrase.

Numerous studies have suggested that agreement or concord accelerates the processing of the target structure, such that the presence of transparent gender marking on one item may facilitate processing of the upcoming gender-marked item (Antón-Méndez et al. 2002; Dussias et al. 2013; Lew-Williams and Fernald 2007). Indeed, transparent gender marking has been shown to facilitate gender agreement processing thanks to access to grammatical gender information via the form-based route (Caffarra et al. 2014; but see Gollan and Frost 2001, for the description of the two-route model). Therefore, concord of markers within the agreement structure may speed up the learning of such a structure. For instance, Alarcon (2011) demonstrated that L2 learners of Spanish benefited from gender marking morphology when processing agreement phrases with transparent nouns as opposed to opaque nouns. This effect was obtained in both, comprehension (in a written gender recognition task) and oral production. Sensitivity to gender agreement marking has been confirmed by eye-tracking (Lew-Williams and Fernald 2010) and neuroimaging research (Gillon-Dowens et al. 2011).

Most studies of gender agreement processing have focused primarily on languages with less fusional morphology, such as Spanish (Alarcon 2009; McCarthy 2008; Montrul et al. 2008; Sagarra and Herschensohn 2010, 2011), Dutch (Lemhöfer et al. 2010) and French (Presson et al. 2014), where gender agreement features only one element (e.g. gender) lacking case declension inflectionally marked on both the adjective and the noun. However, only a few studies have used languages with a complex inflectional system, such as Russian (Brooks et al. 2006, 2011; Kempe et al. 2010; Kempe and MacWhinney 1998), which is considered more morphologically rich due to agreement involving features for number, gender, and case (Lorimor et al. 2008). And even less is known about the acquisition of morphology in these languages under incidental learning conditions (Brooks and Kempe 2013).

### Working Memory and Gender Agreement Processing

According to the Dissociation Hypothesis (Antón-Méndez et al. 2002; Kempen and Hoenkamp 1987), gender is an inherent property of nouns, i.e., it is part of the lemma (the part of a word’s representation that contains syntactic and semantic information). This may impose particular cognitive demands during learning and processing for a speaker whose L1 does not grammatically mark gender. This view is supported by behavioural studies of gender agreement processing in languages such as Spanish (Sagarra and Herschensohn 2010, 2012) and Italian (De Vincenzi and Domenico 1999) and research employing neuroimaging techniques (Barber and Carreiras 2005). For the L2 learner with a relatively poor L1 morphology, acquiring an inflectional morphological pattern is a taxing task (Kempe and MacWhinney 1998) that may induce a cognitive load linked to working memory (WM), an individual’s limited capacity to process and store information during complex cognitive tasks (Baddeley 2003, 2007; Baddeley and Logie 1999; Just and Carpenter 1992). In fact, many studies have demonstrated that WM is relied upon during the processing of gender agreement in L2 learners who already possess some knowledge of a given language (e.g., for Spanish see Sagarra and Herschensohn 2010, 2012)).

Despite the claim that WM is implicated in L2 morpho-syntactic learning and processing in adults (Hummel 2009; Jeeser 2007; Juffs 2004; Michael and Gollan 2005; Miyake and Friedman 1998; Sagarra 2007; Williams and Lovatt 2003), including gender morphology (Keating 2009; Kempe et al. 2010; Sagarra 2007), it is not yet known whether WM plays a major role during incidental learning, a condition in which knowledge seems to be acquired effortlessly (Conway et al. 2011; Kaufman et al. 2010; Tagarelli et al. 2011; Yang and Li 2012). Nevertheless, Kaufman et al. (2010) suggested that WM might indeed be involved in incidental learning, but only during the initial stages. Therefore, it is important to understand the extent to which WM plays a role in the receptive and expressive domains during the incidental acquisition of a cognitively demanding feature of gender agreement in novice learners whose L1 does not grammatically mark gender.

### The Present Study

Research indicates that incidental learning of grammatical structures is based on the processing of specific co-occurring items (Whittlesea and Dorken 1993). This view presupposes that noun-adjective agreement is prone to being acquired via associative-learning mechanisms because of the nature of its morpho-syntactic realization (e.g., two morphological elements occurring closely in the input). Noun-adjective agreement also lends itself to global associative chunk strength (Pothos 2007) and thus has enhanced learnability given the consistency of the paradigm of morphological markers across all lexical items irrespective of their lexical novelty (e.g., the pattern of –*aya* and –*a*, FEM). The present study explored the extent to which receptive and productive knowledge of gender agreement, exemplified by adjective-noun phrases (e.g., *krasn* ***aya*** *shlyap* ***a*** ‘red hat (f)’), in a highly inflectional language (Russian) can be acquired by novice adult learners under incidental learning conditions. In contrast to comprehension (Housen et al. 2005; Rebuschat and Williams 2012; Robinson 1996; Williams 2005; Williams and Evans 1998), the productive aspect of incidental acquisition of grammar has been under-investigated (Brooks and Kempe 2013; Denhovska et al. 2016; Hama and Leow 2010); thus, focusing on both the productive and receptive domains is important. Finally, the mediating role of WM in the retrieval of receptive and productive knowledge acquired under incidental exposure was also addressed.

## Methods

A between-subjects design was employed to explore the effects of the learning condition (incidental learning vs. explicit learning) on *RT*s and accuracy in recognizing and producing the gender agreement pattern. Case (nominative) and number (singular) were kept constant, whereas gender was manipulated by using feminine, masculine and neuter morphological patterns marked on nouns and adjectives within adjective-noun phrases. In the incidental learning condition, we adopted the training paradigm generally accepted in the field to investigate the acquisition of morpho-syntax through incidental exposure. In this paradigm, experimental subjects are asked to focus on meaning and are not informed about the subsequent testing (Rebuschat and Williams 2012; Tagarelli et al. 2011, 2016) in contrast to participants in the explicit learning condition who are informed about testing at the start of the experiment. In the explicit learning condition of the present study, in line with studies showing that an explicit learning condition is generally more effective for L2 grammar knowledge acquisition (DeKeyser 1995; Ellis 2005; Norris and Ortega 2000; Robinson 1997), we provided a metalinguistic explanation of the rule to facilitate a better comparison of the effectiveness of knowledge retention.

### Participants

Forty adult native speakers of English with no previous knowledge or exposure to Russian or any other Slavic language participated in the study. Following Leung and Williams (2011), we excluded participants who had advanced knowledge of a language with grammatical gender. Participants were asked if they speak or have used in educational settings any other language except English. They were further asked to self-report their competency in that language as being “beginner”, “intermediate” or “advanced”. Participants reporting their competency above “beginner” level and those using L2 in bilingual context or educational settings were excluded. The participants were reimbursed with either £ 5 or 10% course credit. All participants were undergraduate and graduate students (3 studying natural sciences, 5 studying social sciences, and 30 studying arts and humanities) at a large university in the UK. The participants included 17 males and 23 females (age range: 18–34).

### Materials

**Fig. 1:**
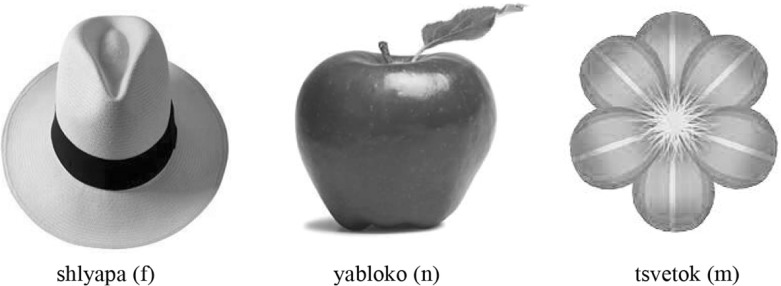
Examples of stimuli used in the pre-training phase

The materials for the study were 37 Russian words: 18 nouns; 18 adjectives; the particle *eto* ‘this’; and semantically corresponding colour pictures depicting inanimate objects of masculine, feminine and neuter genders in Russian (see Fig. 1).^1^

These words were presented in simple Russian sentences, such as *Eto krasn* ***oe*** *yablok* ***o*** ‘This is a red apple’. Each sentence comprised a particle *eto* ‘this is’ followed by the adjective-noun phrase. Russian sentences were transliterated into the Latin alphabet and used along with their English translations written below them. The sentences referred to inanimate objects and contained noun-adjective agreement in the singular nominative case in three genders (masculine, feminine, and neuter), as expressed by the inflectional paradigm of endings on both the noun and the adjective. Only nouns and adjectives that fell into the inflectional paradigm represented in Table 1 were selected.

**Table 1:** Inflectional paradigm of adjectives and nouns in the nominative case for the three genders

Masculine		Feminine		Neuter	
Adjective	Noun	Adjective	Noun	Adjective	Noun
-iy	$\varnothing$	-aya	-a	-oe	-o

The study included three blocks of sentences with (1) feminine gender, (2) masculine gender, and (3) neuter gender agreement. Each block consisted of 12 sentences. The 6 novel nouns of each gender were repeated twice within a block. In addition, 18 adjectives were repeated twice across the blocks in conjunction with a noun of a different gender. Nouns and adjectives were selected on the basis of imageability (e.g., the ability to be easily represented in a picture), and only inanimate nouns were chosen to avoid a potential natural gender bias. Adjectives were disyllabic, and nouns contained 1–3 syllables. Examples of the training sentences are presented in Table 2.

**Table 2:** Example training sentences

Feminine			Masculine			Neuter		
Eto krasnaya shlyapa			Eto beliy parohod			Eto krasnoe yabloko		
This is a red hat			This is a white ferry			This is a red apple		
*Eto*	*krasn-aya*	*shlyap-a*	*Eto*	*bel-iy*	*parohod-Ø*	*Eto*	*krasn-oe*	*yablok-o*
This Ø-cop	red-FEM	hat-FEM	This Ø-cop	white-MASC	ferry-MASC	This Ø-cop	red-NEUT	apple-NEUT

### WM tests

The participants completed two WM tests: operation span and reading span (Unsworth et al. 2005). These tests required them to remember letters in the order presented and either to solve a math operation or to judge the semantic plausibility of an English sentence. In the operation span test during each trial, the participants were presented with one math operation. In the reading span test, they were presented with one sentence at a time, immediately followed by a letter. The math problem/sentence–letter pairs were presented in sets of 3–7 items. After each complete set, the participants were asked to recall the letters in the order presented. Trials consisted of 3 sets of each set size, which ranged from 3 to 7 items. The order of presentation of each set size was random for each participant. The participants were presented with a total of 75 letters and 75 math problems or sentences. The two WM tasks were obtained from the Attention and WM Lab at Georgia Institute of Technology and were used in a number of previous studies (Redick et al. 2012; Turner and Engle 1989).

### Procedure

The participants were tested individually and first completed two WM tests and a pre-training phase, in which they learned Russian vocabulary and performed a test, followed by training and immediate testing stages.

#### Pre-training

The participants learned 37 Russian words by reading the slides on the computer screen at their own pace. Each slide contained a picture with a Russian word and its English translation written below. The participants then completed a vocabulary test on which they had to score no less than 85% to proceed to the training phase. During the vocabulary test, a picture followed by a Russian word written in the Latin alphabet was presented on the computer screen. The participants had to press 1 (“match”) or 2 (“mismatch”) on the keyboard to indicate whether the picture was congruent with the word. The participants received feedback on their performance after each answer in the form of the percentage of correct responses and the word “Correct” or “Incorrect” appearing in the upper left corner of the screen. E-Prime 2 was used to deliver the stimuli (Psychology Software Tools, Pittsburgh, PA). The vocabulary task was included to ensure that the participants would be able to understand the Russian sentences presented during training. Pre-training took 10–15 min depending on the participant as they were memorizing vocabulary items at their own pace.

#### Training

The participants were randomly allocated to one of the two training conditions: the incidental learning condition or the explicit learning condition (20 in each). In the incidental learning condition, the participants were presented with Russian sentences transliterated into the Latin alphabet and their English translations written below. The English translations were included in order to motivate the participants to feel engaged in real language learning. The sentences were presented via E Prime 2; they appeared for 4000 ms on the screen and timed out after this set time for each stimulus. The sentences were presented in blocks: (1) 12 containing agreement in the masculine gender, (2) 12 containing agreement in the feminine gender, and (3) 12 containing agreement in the neuter gender. The order of presentation of the blocks was counterbalanced among the participants, and the presentation of the sentences was randomized. The participants were instructed to read the Russian sentences and translations without performing any additional tasks.^2^ They were not informed about the underlying agreement pattern or the subsequent testing (see Rebuschat and Williams 2012; Tagarelli et al. 2011, 2016 for a similar procedure).

The participants in the explicit learning condition received a metalinguistic explanation of adjective-noun agreement in Russian and were provided with example sentences (see “Appendix” for instructions). They were asked to memorize the agreement rule and were informed that they would be tested on it. The training time for the incidental and explicit learning conditions was the same (15 min).

#### Testing

Immediately after the training, the participants in both conditions completed a Grammaticality Judgement Task (GJT) and a production fill-in-the-blank task. During the GJT, the participants were presented with Russian sentences with their English translations written below. The Russian sentence was either grammatical or ungrammatical because of a gender violation. The participants were informed that the translation was always correct and were instructed to judge the grammaticality of the Russian sentence by pressing the corresponding key on the keyboard. The English translations were always correct, since the violation in the Russian sentences was only at the level of morphology and did not induce any changes in the actual meaning of the sentence. The motivation for including a correct translation was to prompt the participants to focus on morphology. Overall, the participants viewed 28 items, 24 of which were test items and 4 of which were practice items. The test items included in the statistical analyses comprised 12 old items (6 containing correct grammatical agreement and 6 containing agreement violation, for a total of 2 items per gender) and 12 new items unseen during training (6 grammatical and 6 ungrammatical). The old items were familiar sentences containing noun-adjective pairs seen by the participants during training. The new sentences included lexical items that had not been presented during training and contained the same morphological markers as the old items. The new items were created following the same constraints as used for the old items. The ungrammatical items in both the old and new blocks were created such that the adjective contained the endings of a different gender than the one required by the noun. The noun endings were always grammatical. For instance, in an ungrammatical sentence, instead of the ending –*aya* for an adjective agreeing with a feminine noun that has the ending –*a*, it instead had the ending –*iy* (masculine gender) or –*oe* (neuter gender). Thus, the participants were presented with grammatical sentences, such as *Eto bel* ***aya*** *sumka* ‘This is a white bag’ (f), where the ending of the adjective agrees with the noun in gender, and ungrammatical sentences, such as * *Eto krasn* ***oe*** *chashk* ***a*** ‘This is a red cup’, where the ending of the adjective did not agree with the noun in gender:

$$\begin{aligned} \begin{array}{ll@{\quad }l@{\quad }l} ^{*}&{}{ Eto}&{} { krasn}\hbox {-}{} { oe}&{} { chashk}\hbox {-}{} { a}\\ &{}\hbox {This is}&{} \hbox {red-NEUTER}&{} \hbox {cup-FEM}\\ \end{array} \end{aligned}$$

The presentation of stimuli was randomized for each participant. The participants were asked to indicate whether the Russian sentence was grammatical or ungrammatical by pressing the relevant key on the keyboard, and their reaction time and accuracy were recorded during the task.

In the fill-in-the-blank task, the participants were presented with Russian sentences, such as *Eto chist*___ *zerkalo* ‘This is a clean mirror’, with its English translation written below, one at a time. They were asked to fill in the blank by typing in the appropriate inflection for the adjective. The fill-in-the-blank task also comprised the old and new item blocks. All tasks were completed in one 60 min session.

#### Debriefing

At the end of the experiment, the participants in the incidental learning condition were asked whether they had noticed any systematic patterns in the data. They were asked the following: “Did you notice any rules about the Russian sentences?”; their awareness was further probed with “Did you notice any regularities in the sentences or anything about their structure? Can you describe any regularity?”. If the participant was able to verbalize the metalinguistic rule of noun–adjective agreement or simply stated that the ending of one word changed depending on the associated word, they were classified as “aware”. If the participant stated that they did not notice anything, they were classified as “unaware”. See “Appendix” for example answers.

## Results

**Fig. 2:**
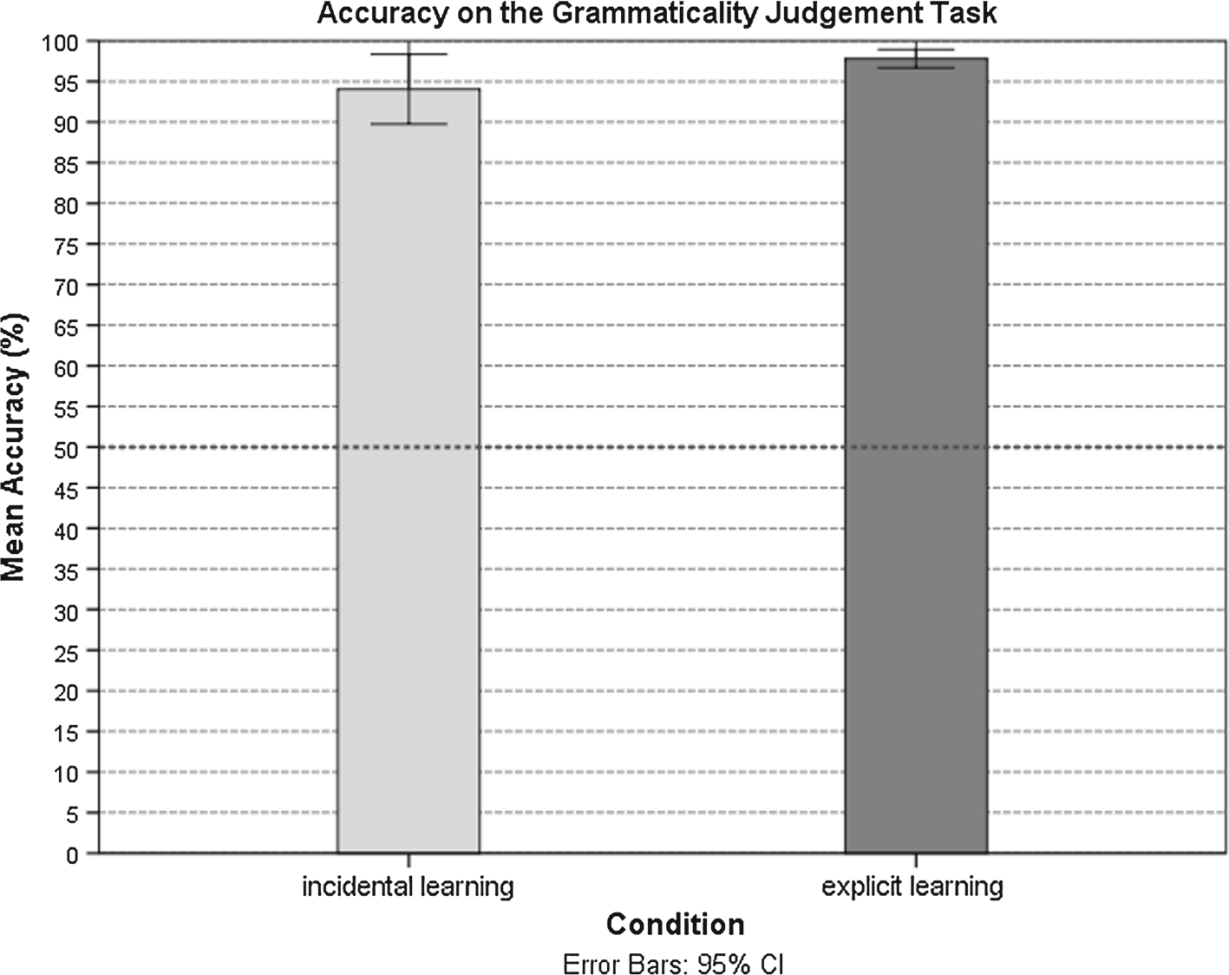
Accuracy on the GJT (%) in the incidental and explicit learning conditions

**Fig. 3:**
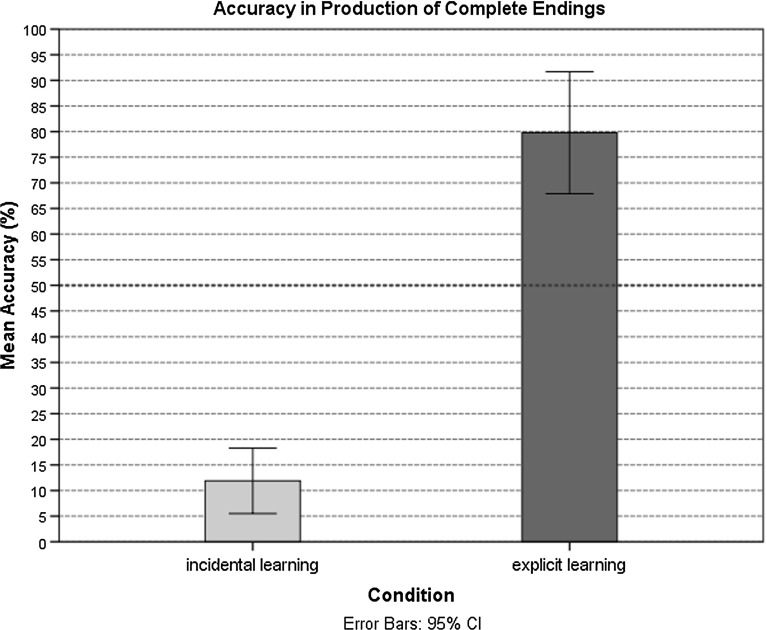
Mean production (%) accuracy in the incidental and explicit learning conditions

The participants in both learning conditions exhibited similar levels of knowledge attainment in receptive domain. Both groups showed a high level of receptive knowledge when they were exposed to the agreement pattern (Fig. 2). Only three participants in the incidental learning condition reported awareness of the gender agreement rule in the post-experiment debriefing. They reported noticing of the underlying grammatical pattern, and they were thus classified as aware. These aware participants showed higher knowledge levels in production than those who did not report awareness of the agreement regularity; their performance was 100, 93, and 93%, respectively. These participants were excluded from the analyses, and their data were substituted with the data for three additionally recruited participants who did not show awareness of the regularity underlying the pattern. Generally, performance in production in the incidental learning conditions was poor, while participants in the explicit learning condition performed at a level above chance (Fig. 3).

The data were analysed using logistic and linear regression models in R, version 3.2.3, by applying a generalized linear model (GLM) using the R Commander software package (R Development Core Team 2015). We checked for normality and homogeneity by visual inspections of the plots of residuals against the fitted values. Throughout the paper, MCMC-estimated p-values that are considered significant at the $\upalpha = 0.05$ level are presented. We used a backward model selection procedure that started with a full model including all parameters and excluding them one at a time. ANOVA function was used to determine whether each parameter significantly improved the model (Baayen et al. 2008). When fitting the model, all fixed effects of theoretical interest were retained in the model, even if they were non-significant. The model selection procedure and a summary of model coefficients is represented in Table 3.

**Table 3:** Model selection

Predictor	AIC	BIC	Pr $(>\hbox {Chisq})$
Condition	322	331	.027
Block (old vs. new)	314	329	.005
Grammaticality	314	333	.118
Gender	316	345	.255
Operation span	317	351	.953
Reading span	320	363	.272
Block $\times$ gender	323	376	.320
Condition $\times$ grammaticality	319	363	.294
Condition $\times$ block	314	361	.004

*Full model:* Condition, Block, Grammaticality, Gender, Operation Span, Reading Span, Block $\times$ Gender, Condition $\times$ Grammaticality, Condition $\times$ Block

**Table 4:** Analysis of GJT accuracy and RTs

Factor	Estimate	Standard error	Wald z	*p*
*Accuracy*				
(Intercept)	2.18	1.00	2.17	.30
Condition				
Incidental learning versus explicit learning	1.53	.51	2.97	.003*
Gender				
Feminine versus masculine	.43	.38	1.12	.26
Feminine versus neuter	.45	.46	.97	.33
Grammaticality				
Grammatical versus ungrammatical	.59	.35	1.66	.09
Block				
New versus old items	.31	.60	.52	.61
Operation Span	.00	.02	.07	.93
Reading Span	.02	.02	1.08	.28
Incidental learning $\times$ old items	2.18	.81	2.69	.007**

Factor	Estimate	Standard error	t value	*p*
*RT*s				
(Intercept)	2175.55	161.29	13.49	<.001
Condition				
Incidental learning versus explicit learning	−344.25	77.49	−4.44	<.001
Gender				
Feminine versus masculine	83.20	61.25	1.36	.17
Feminine versus neuter	114.07	67.80	1.68	.09
Grammaticality				
Grammatical versus ungrammatical	93.63	52.67	1.77	.08
Block				
New versus old items	−27.79	74.73	−.37	.71
Operation Span	−8.46	3.14	−2.67	.007**
Reading Span	−0.22	104.63	−.07	.94
Incidental learning $\times$ old items	−2.59	104.63	.03	.98

### Receptive Knowledge Acquisition

A logistic regression GLM model was run to analyse the accuracy of the GJT of the agreement pattern. Condition (incidental learning, explicit learning), Gender (feminine, masculine, neuter), Grammaticality (grammatical, ungrammatical), Block (new, old items), Operation span score and Reading span score were included in the model as fixed effects, along with the interaction Condition $\times$ Block; Subject was included as a random effect.

**Fig. 4:**
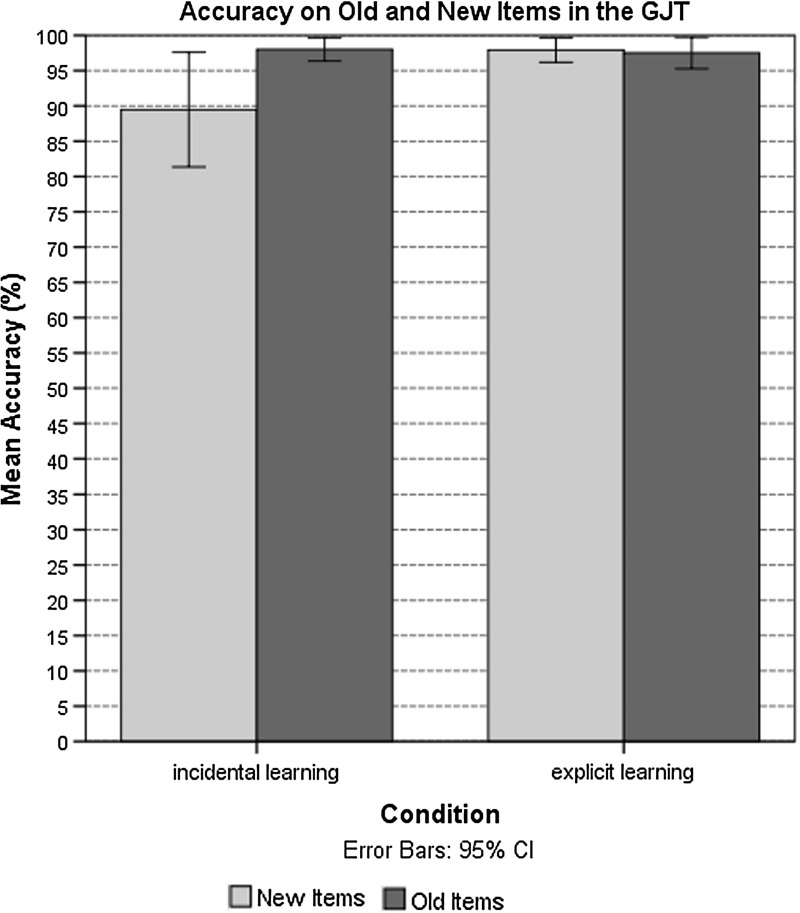
Accuracy in GJT of old and new items (%) in the incidental and explicit learning conditions

There was a significant main effect for learning condition: the participants in the incidental learning condition $(M = 94.05\%, { SD} = 9.17\%; \beta = 1.53, { Wald\,z} = 2.97, { SE} = .51, p = .003)$ recognized the agreement pattern less accurately than the participants in the explicit learning condition $(M =98.00\%,{ SD} = 2.46\%)$. There were no effects of WM or gender (see Table 4). However, the overall identification of items showing violations compared to grammatical items approached significance, with violations being identified more accurately $(\beta =.59, { Wald\,z} = 1.66, { SE} = .35, p = .09)$. Additionally, the participants in the incidental learning condition scored more accurately on the trained items than on the untrained items $(\beta = 2.18,{ Wald\,z} = 2.69, { SE} = .81, p = .007)$ (Fig. 4).

**Fig. 5:**
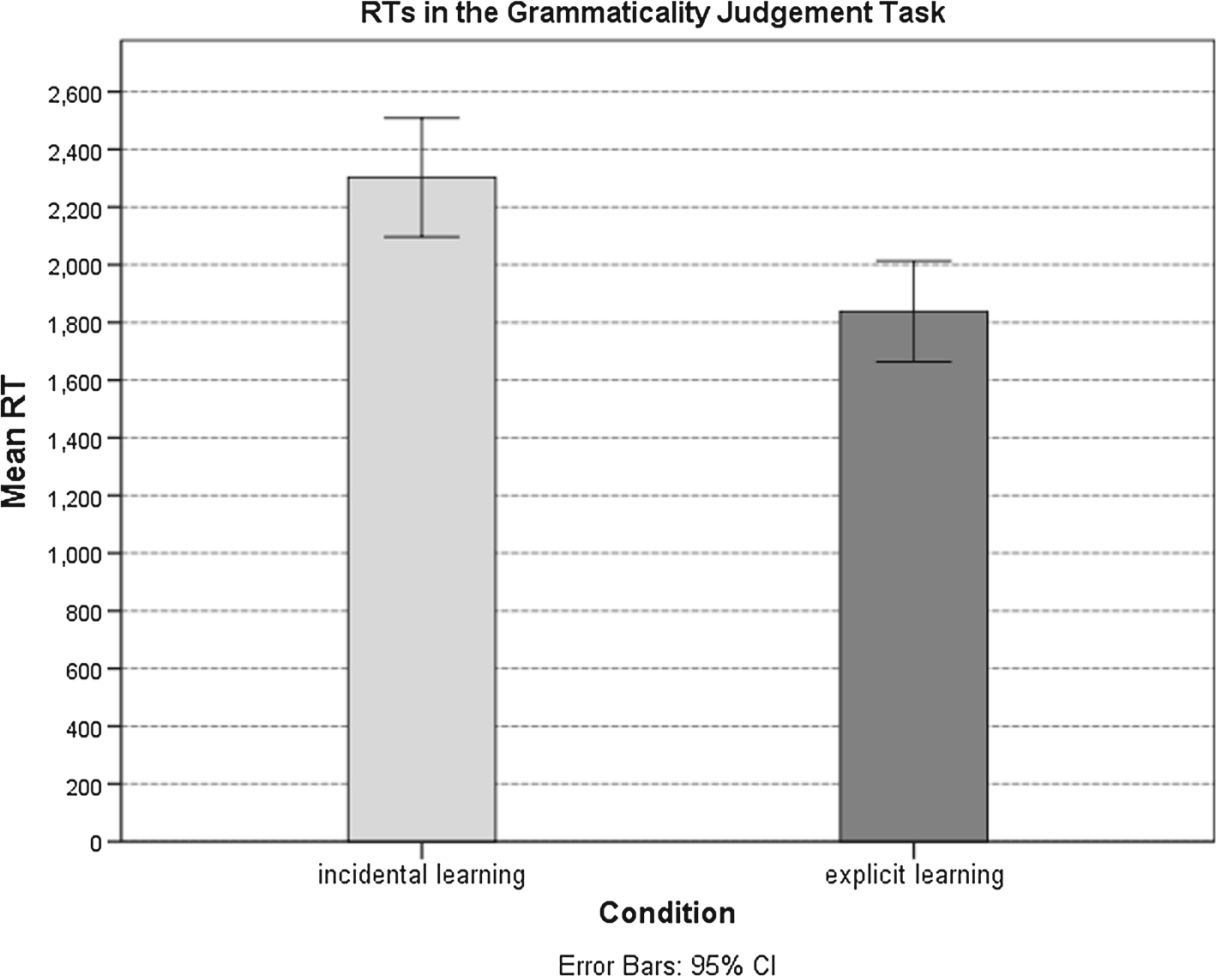
Mean reaction times on the GJT in the incidental and explicit learning conditions

A linear regression model with the same variables was run to analyse the participants’ response times during recognition of the agreement pattern. The results indicated that the participants in the incidental learning condition responded significantly more slowly than the participants in the explicit learning condition $(\beta = -344.25, { t\,value }= -4.44, { SE} = 77.49,p < .001)$. Longer *RT*s for the ungrammatical items were observed $(\beta =93.63, { t\,value }= 1.77, { SE} = 52.67, p = .08)$; however, no difference in *RT*s were found for familiar (old) and unfamiliar (new) items $(\beta = -27.79, { t\,value }= -.37, { SE} = 74.73, p = .71)$. The mean response times are presented in Fig. 5.

### Productive Knowledge Acquisition

A logistic regression model with the following variables was run to analyse accuracy in production: Condition (incidental learning, explicit learning), Gender (feminine, masculine, neuter), Block (new, old items), Operation span score and Reading span score were included as fixed effects, along with the interaction Condition x Block; Subject was included as a random effect.

**Fig. 6:**
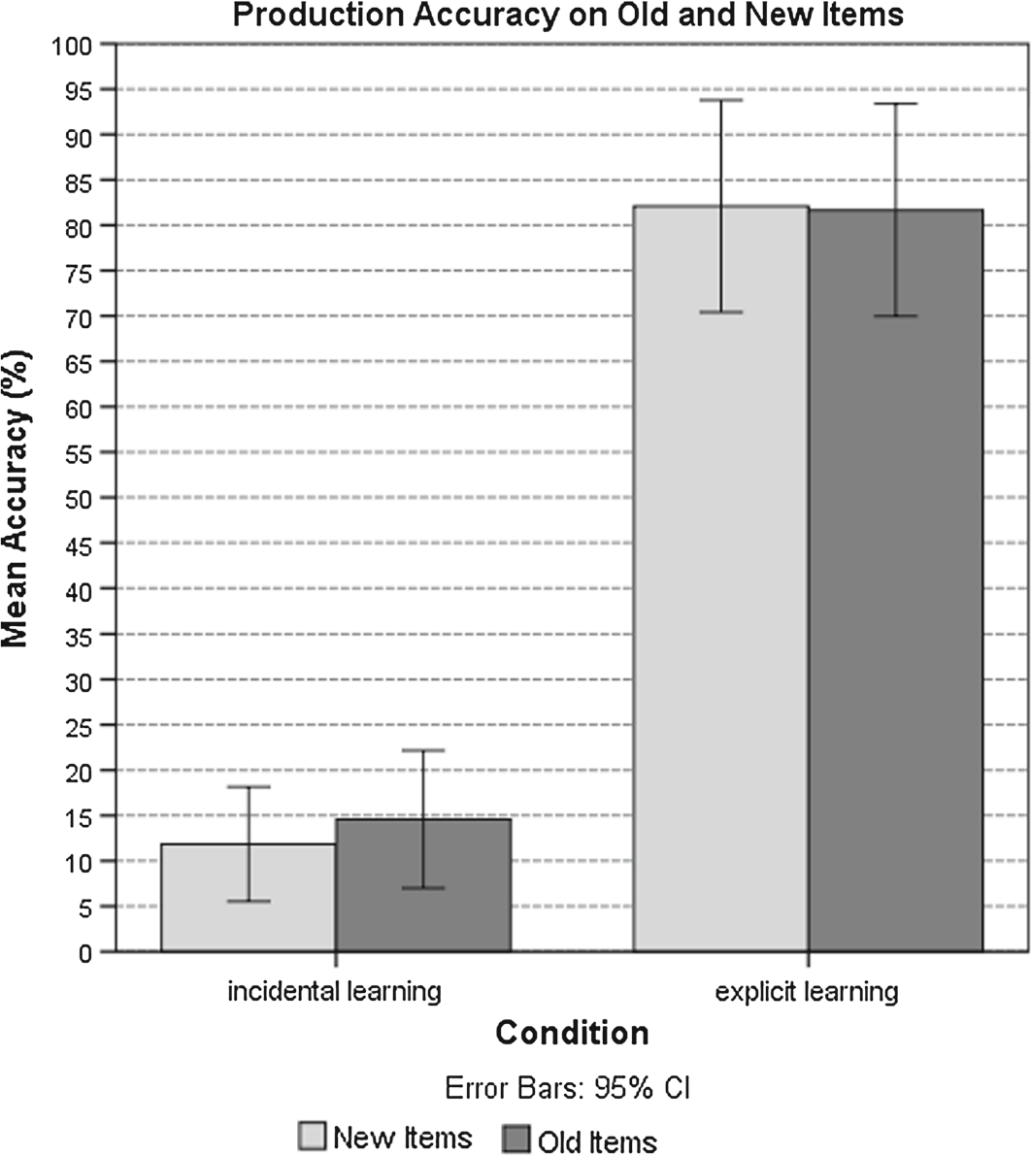
Production accuracy (%) of old and new items in the incidental and explicit learning conditions

The analysis revealed that the participants in the incidental learning condition $(M = 11.90\%, { SD} = 14.04\%; \beta = 3.93, { Wald\,z} = 12.11, { SE} = .30, p < .001)$ recalled the correct form of the appropriate adjectival ending significantly less accurately than the participants in the explicit learning condition $(M = 79.79\%,{ SD} = 25.44\%)$ (see Table 5). A WM effect in the retrieval of appropriate morphological markers was found, as indicated by both the operation $(\beta = .03, { Wald\,z} = 2.61, { SE} = .01, p = .01)$ and reading span tasks $(\beta = .03, { Wald\,z} = 2.61, { SE} = .01, p = .01)$. The results showed no difference in the accuracy of recall of the appropriate ending between the old and new item blocks in either learning condition (Fig. 6). We found an effect of gender; items with masculine $(\beta = 1.12, { Wald\,z} = 4.67, { SE} = .24, p < .001)$ and neuter $(\beta = 1.31, { Wald\,z} = 5.41, { SE} = .24, p < .001)$ genders were produced significantly more accurately than morphological agreement markers of the feminine gender.

**Table 5:** Analysis of production accuracy

Factor	Estimate	Standard error	Wald z	*p*
*Production Accuracy*				
(Intercept)	−10.24	.86	−2.54	<.001
Condition				
Incidental learning versus explicit learning	3.93	.30	12.11	<.001
Gender				
Feminine versus masculine	1.12	.24	4.67	<.001
Feminine versus neuter	1.31	.24	5.41	<.001
Block				
New versus old items	.61	.63	.98	.33
Operation Span	.03	.01	2.61	.01
Reading Span	.33	.01	2.61	.01
Condition $\times$ block	.03	.38	−.85	.40

### Working Memory

A series of two-tailed Pearson correlations were carried out for both WM measures (the operation and reading span task scores) in order to better understand the role of WM in receptive and productive knowledge acquisition under the incidental learning condition. The results demonstrated a moderate positive correlation with WM, as measured by the reading span $$(r = .53, p = .01)$$ and the operation span $$(r = .49, p = .03)$$ for production accuracy. However, a null effect of WM was found for both the GJT accuracy (reading span: $$r = .21, p = .37$$; operation span: $$r = .19, p = .43)$$ and *RT*s (reading span: $$r = .14, p = .57$$; operation span: $$r = -.14, p = .57)$$ (see Table 6 for more detailed results).

**Table 6:** Correlations with WM in the incidental learning condition

OS total		OS score		RS total		RS score	
*r*	*p*	*r*	*p*	*r*	*p*	*r*	*p*
*GJT accuracy*							
.19	.43	.20	.39	.21	.37	.18	.45
*RT*s							
−.14	.57	.10	.69	.14	.57	.13	.60
*Production accuracy*							
.49	.03*	.57	.006**	.53	.01*	.56	.009**

*$$p<.05$$, **$$p<.01$$, ***$$p<.001$$

*NB: *OS and RS total refers to the number of letters recalled in the correct order;

OS and RS score refers to the number of letters recalled irrespective of their order

## Discussion

The aim of the current study was to investigate the extent to which adults can incidentally acquire receptive and productive knowledge of a grammatical feature not present in their L1 (gender agreement inflectionally marked in Russian adjective-noun combinations) and whether such learning is predicted by WM. Only transparent gender markers were used for noun-adjective agreement. The findings indicated that speakers of a language that lacks grammatical gender can reach a high level of accuracy in judging the grammaticality of a noun-adjective agreement pattern in a new language without instruction, after a very limited amount of exposure to the morphological regularity in the input.

This finding is in line with research showing that adults generally performed at levels above chance in the comprehension of semi-artificial language grammars (Williams 2005; Leung and Williams 2011; Rebuschat and Williams 2012). It also demonstrates that to some extent post-puberty learners are able to learn new grammatical features not present in their L1 (Schwartz and Sprouse 1996; Leung 2005; White et al. 2004). As such, these features can be accessed via procedural learning mechanisms: the mechanisms of tracking statistical regularities and detecting associations between the two elements in the input (the ending of a noun and that of an adjective) without instruction and intention, as indicated by the extensive research on statistical learning in language acquisition (Conway et al. 2011; Fiser and Aslin 2001; Kim et al. 2009; Kidd 2012; Misyak and Christiansen 2012; Reber 1967; Saffran 2003; Saffran et al. 1996). Moreover, the findings of the present study suggest that grammatical agreement might be different from other grammatical structures in terms of susceptibility to incidental learning mechanisms. Studies comparing the learning of grammatical structures such as negation and the passive voice in L2 (Housen et al. 2005) have typically found an advantage for the explicit learning condition (DeKeyser 1995; Norris and Ortega 2000; Robinson 1996). Overall, our findings on the GJT are in line with the work of Morgan-Short et al. (2010), who showed that adults are not only able to attain knowledge of gender agreement in an artificial language but also exhibit similar levels of knowledge intake in incidental and explicit learning conditions.

When individuals learn incidentally, extracting knowledge about gender agreement-marking regularities from the input appears to be piecemeal and based on memorization. As shown by our findings on the GJT, learners performed better on the familiar items (items seen during training) than on the unfamiliar items. At the same time, one could argue that such learning of agreement dependencies is fostered by various facilitating factors available to the learner, including pattern consistency. As indicated by Taraban (2004), learners can notice grammatical patterns and draw generalizations using semantic, morphological and phonological cues available in the input when they are learning under incidental exposure. In the present study, our participants successfully detected form-based violations signalling the wrong gender within the consistent pattern of transparent morphological markers (e.g., with the “*-oe –o*” pattern for the neuter gender, the “-*aya* –*a*” pattern for the feminine gender, and the “-*iy – *Ø” pattern for the masculine gender). The assumption that transparent marking can serve as a cue to gender has been supported by numerous studies of gender agreement processing in such languages, as Spanish (Alarcon 2011; Caffarra et al. 2014) and Russian (Kempe and Brooks 2001, 2008). In fact, Kempe and Brooks (2008) indicated that if attention of L2 learners is not directed to the regularity in the morphological pattern, transparent markers are necessary in order for the grammatical category to be learnable. They further suggested that such marking in the nominative case allows for the L2 learner of Russian morphology to effortlessly infer the gender category membership. Thus, we did not find any gender-related differences in the GJT, as patterns of all three genders were recognized equally accurately. Interestingly, however, learners tended to produce shorter endings with higher accuracy [e.g., –*oe* (n), -*iy* (m)] than longer endings [-*aya* (f)], possibly as a result of the larger cognitive load involved. Similar results regarding more erroneous production of feminine gender morphology in Russian were obtained by Brooks et al. (2006).

Overall, to process agreement, the learner must detect gender marking on the governing word (noun) and attach the relevant marker to the adjective. When gender marking on the noun is opaque, research has shown that learners use adjectival inflections as a cue (Taraban and Kempe 1999). Above-chance performance—without reporting awareness about gender—in the GJT confirms that novice learners in this study were guided by the formal features of gender, knowledge of which was obtained during training under incidental exposure. While there is evidence that late L2 learners exhibit difficulties in both lexical gender assignment and syntactic computation of gender agreement, especially if their L1 does not mark gender (Franceschina 2005), a number of studies have demonstrated that learners are susceptible to distributional information in the input and are able to detect formal cues of grammatical gender (Hernandez et al. 2004; Taft and Meunier 1998) and of agreement (Bates et al. 1996; Gollan and Frost 2001). This finding, however, might apply to the receptive side only. The production of such markers, at least at the very initial stages of learning and with very limited exposure—as was the case in the present study, appears to require reliance on explicit knowledge about grammatical gender at the lexical level. This assumption is supported by the present study finding that only learners who were provided with metalinguistic instruction (the explicit learning condition) and learners who reported awareness (three individuals in the incidental learning condition) performed at a level above chance on the production task. An important role of metalinguistic awareness in the production of complex morphological systems was also pointed out by Brooks and Kempe (2013), who focused on the acquisition of Russian case-marking under incidental exposure.

Such a discrepancy between expressive and receptive performance can be explained by the two-route hypothesis of gender information processing. This model suggests that grammatical gender is retrieved both via the form-based route, where it is accessed through formal cues realized via gender agreement dependencies, and it is also recovered by accessing abstract knowledge about gender of a given lexical item from the mental lexicon (Caffarra et al. 2014; Gollan and Frost 2001). During comprehension, learners potentially access the form-based route. In transparently marked items, the morphological form serves as a reliable cue about the grammatical gender of inanimate nouns (Caffarra et al. 2014); thus, it facilitates efficient gender information acquisition and automatic retrieval of the acquired knowledge, as indicated by the strong learning effect and null correlation with WM observed in the receptive domain. As demonstrated by studies using grammaticality judgements in different languages, such as French, Spanish, Italian, and Hebrew, formal gender-marking cues allow rapid access to grammatical gender information (Bates et al. 1996; Gollan and Frost 2001; Taft and Meunier 1998).

However, our findings suggest that during production, possessing lexical knowledge of the noun’s gender is a prerequisite for the successful production of gender agreement morphology (Hopp 2016; Lemhöfer et al. 2014). During the very initial stages of learning, such knowledge is developed and accessed via conscious effort. The cognitive demands accompanying this process may have been captured by the association with WM measures in the present study. For instance, neurocognitive evidence indicates that developing awareness is accompanied by neural activity in the brain that is observed before the acquired knowledge can be verbalized (Rose et al. 2011). Rogers et al. (2016), who investigated the incidental acquisition of Czech morphology, also suggested that a production task by its very nature promotes the formation of conscious knowledge.

Overall, researchers believe that gender processing in adult learners is more challenging, due to the weaker and less stable nodes of noun’s gender in the mental lexicon (Hopp 2016). In advanced learners, abstract gender representations may be more robust, and thus retrieved more automatically from the mental lexicon. A longitudinal study over multiple sessions may shed light on the issue of how lexical gender representations develop and are accessed during production. In support of the assumption that conscious effort is needed at initial stages Brooks and Kempe (2013), who conducted production testing after six sessions of incidental exposure to the Russian case-marking system, found a null WM effect, which might indicate the automaticity of knowledge retrieval at that stage. Similarly, Kaufman et al. (2010) proposed that WM is relied upon only during the initial stages of knowledge acquisition under incidental learning conditions.

Another explanation for the present study finding of a correlation between WM and production accuracy is the involvement of different types of memory during productive knowledge retrieval. The correlation with the operation span may suggest that, instead of simply activating automatic knowledge, the learners had to perform some form of knowledge manipulation. Research suggests that during language processing, different WM and executive functions are engaged (Linck et al. 2013), but little is known about what exactly these functions are and what their roles may be. The correlation with operation span scores may suggest recourse to processes of maintaining, updating and shifting (Miyake et al. 1999; Miyake and Friedman 2012). This indexes effortful learning and a learner’s attempt to “make sense” of the knowledge initially tapped implicitly under incidental learning conditions. In contrast, the correlation with reading span scores found in the incidental learning conditions are in line with assumptions posited by MacDonald and Christiansen (2002) that the reading span tasks tap into experience-based language processing skills in addition to memory and may thus indicate the occurrence of procedural processes. Future research should examine more closely the issue of different types of memory involved in incidental learning.

Overall, despite its limitations as a controlled lab-based experiment, this study is a timely contribution to the incidental learning research and findings obtained on artificial language learning, due to using more ecologically valid stimuli (Erickson and Thiessen 2015), and due to the investigation of the very initial stages of the emergence of grammatical knowledge. Extending the study to other natural languages with complex morphology and conducting a longitudinal study in more naturalistic settings would provide a better understanding of real-world incidental L2 learning.

## Appendix 1: Stimuli Used in the Current Study

**Table:** Nouns of three genders used in the vocabulary learning test

Feminine	Masculine	Neuter
shlyapa – hat	zont – umbrella	zerkalo – mirror
chashka – cup	dom – house	pero – feather
tarelka – plate	tsvetok – flower	derevo – tree
sumka – bag	noj – knife	yabloko – apple
vilka – fork	parohod – ferry	palto – coat
svecha – candle	galstuk – tie	steklo – piece of glass

*Adjectives*

seriy – grey

siniy – blue

krasniy – red

beliy – white

jeltiy – yellow

cherniy – black

uzkiy – narrow

stariy – old

noviy – new

chistiy – clean

gryazniy – dirty

nizkiy – short

dlinniy – long

tonkiy – thin

krupniy – big

tonkiy – thin

tolstiy – thick

temniy – dark

*Training Sentences*

- Feminine block
  - Eto sinaya shlyapa – This is a blue hat
  - Eto staraya chashka – This is an old cup
  - Eto chistaya tarelka – This is a clean plate
  - Eto krupnaya sumka – This is a big bag
  - Eto gryaznaya vilka – This is a dirty fork
  - Eto krasnaya shlyapa – This is a red hat
  - Eto jeltaya chashka – This is a yellow cup
  - Eto novaya tarelka – This is a new plate
  - Eto belaya sumka – This is white bag
  - Eto seraya vilka – This is a grey fork
  - Eto tonkaya svecha – This is a thin candle
  - Eto tolstaya svecha – This is a thick candle
- Masculine block
  - Eto siniy tsvetok – This is a blue flower
  - Eto jeltiy tsvetok – This is a yellow flower
  - Eto beliy parohod – This is a white ferry
  - Eto seriy noj – This is a grey knife
  - Eto maliy parohod – This is a small ferry
  - Eto cherniy zont – This is a black umbrella
  - Eto dlinniy noj – This is a long knife
  - Eto nizkiy dom – This is a short house
  - Eto maliy dom – This is a small house
  - Eto svetliy zont – This is a light umbrella
  - Eto uzkiy galstuk – This is a narrow tie
  - Eto temniy galstuk – This is a dark tie
- Neuter block
  - Eto krupnoe yabloko – This is a big apple
  - Eto chistoe zerkalo – This is a clean mirror
  - Eto gryaznoe zerkalo – This is a dirty mirror
  - Eto krasnoe yabloko – This is a red apple
  - Eto novoe palto – This is a new coat
  - Eto chernoe palto – This is a black coat
  - Eto dlinnoe pero – This is a long feather
  - Eto nizkoe derevo – This is a short tree
  - Eto staroe derevo – This is an old tree
  - Eto uzkoe pero – This is a narrow feather
  - Eto tonkoe steklo – This is a thin piece of glass
  - Eto tolstoe steklo – This is a thick piece of glass

## Appendix 2: Instruction Script for the Explicit Learning Condition

In Russian, adjectives grammatically agree with nouns in gender, case and number and this agreement is conveyed by inflections. Nouns can be of masculine, feminine or neuter gender. The adjective has to change its inflection in order to agree with the noun depending on the noun’s gender.

For instance, in the nominative case, singular, if the noun is of masculine gender, it is indicated by zero ending and the noun normally ends with a consonant. The adjective, which agrees with this noun will have the ending –*iy. *For example, *Eto sin* ***iy*** *tsveto* ***k***_ ‘This is a blue flower’ or *Eto chern* ***iy*** *zon* ***t***_ ‘This is a black umbrella’.

Nouns of feminine gender usually end with –*a*. Therefore, the adjective, which agrees with a feminine noun, will have the ending *–aya*. For example, *Eto bel* ***aya*** *sumk* ***a*** ‘This is a white bag’ or *Eto krasn* ***aya*** *shlyap* ***a*** ‘This is a red hat’.

Finally, if the noun is of neuter gender, it will have ending –*o*. The adjective that agrees with a noun of a neuter gender, will have to adopt the inflection *–oe. *For example, *Eto krasn* ***oe*** *yablok* ***o***‘This is a red apple’ or *Eto chern* ***oe*** *palt* ***o*** ‘This is a black coat’.

## Appendix 3: Responses of Aware and Unaware Participants in the Incidental Learning Condition during Debriefing

**Table:**

Level of awareness	Response
Aware	(1) Endings were similar
	(2) Gender rules
	(3) If one word ended in $-o$ the other one ended in *–oe*; if one word ended in $-a$ the other one ended in $-a$ too
Unaware	(1) Nothing
	(2) Did not notice anything
	(3) No rules noticed

## Footnotes

1 Gender labels were not provided on the training materials.

2 No additional task was included as we wanted to approximate the training to the immersion-like settings as close as possible.
